# Genome-wide DNA methylation analysis identifies kidney epigenetic dysregulation in a cystinosis mouse model

**DOI:** 10.3389/fcell.2025.1638123

**Published:** 2025-08-21

**Authors:** M. N. Rossi, A. Ciolfi, V. Matteo, L. Pedace, C. Nardini, E. Loricchio, I. Caiello, F. Bellomo, A. Taranta, E. De Leo, M. Tartaglia, F. Emma, F. De Benedetti, E. Miele, G. Prencipe

**Affiliations:** ^1^ Laboratory of Rheumatology, Bambino Gesù Children’s Hospital, IRCCS, Rome, Italy; ^2^ Department of Science, University of Roma Tre, Rome, Italy; ^3^ Molecular Genetics and Functional Genomics, Bambino Gesù Children’s Hospital, IRCCS, Rome, Italy; ^4^ Hematology/Oncology and Stem Cell Transplantation, Bambino Gesù Children’s Hospital, IRCCS, Rome, Italy; ^5^ Laboratory of Nephrology, Bambino Gesù Children’s Hospital, IRCCS, Rome, Italy

**Keywords:** cystinosis, DNA methylation, solute carrier genes, kidney disease, proximal tubular epithelial cells

## Abstract

**Introduction:**

Nephropathic cystinosis is a rare genetic disorder characterized by cystine accumulation in lysosomes that causes early renal dysfunction and progressive chronic kidney disease. Although several metabolic pathways, including oxidative stress and inflammation, have been implicated in the progression of renal parenchyma damage, the precise mechanisms driving its progression are not fully understood. Recent studies suggest that epigenetic modifications, particularly DNA methylation (DNAm), play a critical role in the development of chronic kidney disease. We hypothesized that epigenetic dysregulation may contribute to the progression of kidney disease in cystinosis.

**Methods:**

To investigate this, we conducted genome-wide DNAm analyses on kidneys harvested from 6-month-old wild type (WT) and *Ctns* ^*−/−*^ mice, a well-established model of cystinosis.

**Results:**

Our analysis revealed extensive DNAm alterations in cystinotic kidneys, characterized by a significant hypermethylation profile. Interestingly, the majority of differentially methylated CpG sites were located within gene bodies and to a lesser extent in promoter and enhancer regions. Methylation changes were primarily found in genes and pathways crucial for kidney function, particularly those related to the physiology of the proximal tubules. Importantly, DNAm changes correlated with changes in gene expression, as validated by qPCR analyses of key genes. Furthermore, *in vitro* treatment of human proximal tubular epithelial cells with the demethylating agent decitabine resulted in the upregulation of critical transporter genes, suggesting a potential therapeutic approach.

**Conclusions:**

These findings underscore the role of epigenetic regulation in the progression of kidney damage in cystinosis and suggest that DNAm could serve as a promising target for novel therapeutic strategies.

## Introduction

Nephropathic cystinosis is a rare lysosomal storage disorder presenting early in life with renal Fanconi syndrome that progressively leads to chronic kidney disease (CKD). The disease is caused by variants in the *CTNS* gene, which encodes the lysosomal cystine/H+ symporter cystinosin. Progressively, cystine and cystine crystals accumulate in all tissues, causing a multisystemic disease. To date, the only approved treatment for cystinosis is cysteamine, a sulfhydryl compound that reduces disulfide bonds, allowing cystine clearance from lysosomes. Cysteamine significantly slows the progression of kidney failure but, unfortunately, cannot prevent it, suggesting that cystine accumulation is not the sole mechanism driving kidney failure. Other proposed mechanisms include increased oxidative stress, mitochondrial function impairment, enhanced apoptosis, and abnormal autophagy (reviewed in Sur et al., 2024).

Epigenetic modifications, particularly DNA methylation (DNAm) at cytosine-phosphate-guanine (CpG) sites, have emerged as significant contributors to kidney parenchymal damage and CKD (Wanner and Bechtel-Walz, 2017). Large-scale studies have highlighted the importance of DNAm in preserving kidney health. For example, DNAm has been shown to explain a higher portion of kidney disease heritability than gene expression in high-throughput DNA methylation and transcriptomic profiling of 506 human kidneys (Liu et al., 2022). Similarly, meta-analysis of epigenome-wide association studies performed in ∼35,000 adults has identified multiple CpG sites with causal links to kidney function, further supporting the role of epigenetic regulation in kidney health and disease (Schlosser et al., 2021).

DNAm is a reversible process that modulates gene expression without modifying the DNA sequence. Specifically, DNA methylation in the promoter and in the first exon/intron regions can suppress gene expression by recruiting transcriptional repressors or by preventing binding of transcription factors (Anastasiadi et al., 2018; Bird, 1986).

Despite the growing understanding of these processes, the mechanisms underlying epigenetic dysregulation in kidney disease remain incompletely defined. Several lines of evidence support a link between the epigenome and cellular metabolism. In particular, recent data point to a critical crosstalk between oxidative stress, inflammation and epigenetic regulation, which may further contribute to the metabolic and inflammatory dysfunction observed in CKD (Liu et al., 2022; Stenvinkel et al., 2007). Since oxidative stress and inflammation have been shown to play a pathogenic role in cystinosis (Prencipe et al., 2014; Rossi et al., 2019; Vaisbich et al., 2011), we have hypothesized that epigenetic dysregulation might also contribute to CKD progression. To investigate this hypothesis, we conducted a comprehensive methylation analysis on kidneys harvested from *Ctns* ^*−/−*^ mice, a well-established model for cystinosis (Nevo et al., 2010).

## Materials and methods

### Mice care and procedures

The C57BL/6 *Ctns* ^−/−^ mice were kindly provided by Prof. Corinne Antignac (Nevo et al., 2010) and housed alongside their wild-type (WT) C57BL/6 counterparts. Animal care and experimental procedures complied with the European 2010/63/EU on the protection of animals used for scientific purposes and were approved by the Italian Ministry of Health (authorization number 898/2017-PR). Female mice were sacrificed at 6 months of age, and their kidneys were promptly dissected, snap-frozen, or processed in paraffin for further analysis.

### DNA methylation analysis

Genomic DNA was extracted from paraffin-embedded mouse kidney using standard techniques. We adopted a protocol that mitigates fixation artifacts and restores the quality and integrity of degraded DNA from FFPE samples, making it compatible with subsequent steps in the Infinium workflow (Infinium FFPE DNA Restoration Solution, Illumina, cod. WG-321-1002).

DNAm profiling was performed using the Illumina Infinium Mouse Methylation BeadChip array and 500 ng DNA as input material, according to the manufacturer’s protocol. BeadChip processing was performed using an Illumina iScan microarray platform.

Data analysis was performed through an in-house pipeline using the R programming language (v.4.1.2), mainly based on the ENmix package (v.1.30.03) for importing IDAT files, performing data quality control, and correcting for background and dye bias noise (Xu et al., 2021). Methylation levels (beta-values) were converted to M-values, which were used to perform linear regression modelling using empirical Bayes moderated t-statistic (Limma package [v.3.50.3] (Ritchie et al., 2015) corrected for false discovery rate (Benjamini-Hochberg’s FDR) and to identify differentially methylated probes (DMPs),which were considered significant if methylation difference was >15% and FDR was <0.01. Normalized beta-values for each sample were compared by means of multidimensional scaling (MDS) and Hierarchical clustering (HC) analyses, considering the pair-wise Euclidean distances between samples. Differentially methylated regions (DMRs) were determined using ipdmr function with seed = 0.01 (Xu et al., 2021).

Gene-set enrichment analysis on differentially methylated genes was carried out by means of Enrichr-KG and panther tools using default parameters (Evangelista et al., 2023; Mi and Thomas, 2009). Annotation of CpG sites was defined as follows: N_Shelf: Genomic coordinates of a CpG Island North Shelf, where the array has targeted a CpG within the shelf. The definition of a North Shelf is the region 4,000–2000 base pairs upstream of a CpG Island start site; N_Shore: Genomic coordinates of a CpG Island North Shore, where the array has targeted a CpG within the shore. The definition of a North Shore is the region 2000–0 base pairs upstream of a CpG Island start site; CpG_Island: Genomic coordinates of a CpG Island, where the array has targeted a CpG within the island. CpG Islands are regions greater than 200 base pairs in length with GC content of 50% or greater and have a ratio of >0.6 for the observed number of CG dinucleotides to the expected number considering the total number of G and C bases in the genome segment; S_Shelf: Genomic coordinates of a CpG Island South Shelf, where the array has targeted a CpG within the shelf. The definition of a South Shelf is the region 4,000–2000 base pairs downstream of a CpG Island start site; S_Shore: Genomic coordinates of a CpG Island South Shore, where the array has targeted a CpG within the shore. South Shore indicates the region 2000–0 base pairs downstream of a CpG Island start site.

### RNA isolation and quantitative real-time PCR

Total RNA was extracted from whole mouse kidney tissues snap-frozen immediately after mice sacrifice, using Trizol reagent (Ambion). Total RNA was also extracted from conditionally immortalised proximal tubular epithelial cells (ciPTEC), collecting cells directly in Trizol reagent (Ambion). cDNA was obtained using the Superscript Vilo kit (Invitrogen). Real-time PCR assays were performed using TaqMan Universal PCR Master mix (Applied Biosystems) and the following gene expression assays: mouse *Slc7a7*, *Slc4a4*, *Cubn, Aqp1*, *Hnf1a*, *Hnf4a*; human *SLC7A7*, *AQP1*, and *CUBN*. Gene expression data were normalized using mouse *Hprt1* or human *HPRT1* (Applied Biosystems) as endogenous controls. Data are expressed as arbitrary units (AU), determined using the 2^−ΔΔCT^ method.

### Cell culture and decitabine treatment

A control and a cystinotic (carrying the 57 kb deletion of the *CTNS* gene) conditionally immortalized PTEC lines (ciPTEC) were kindly provided by Prof. Elena Levtchenko and cultured as described in Wilmer et al. (2005). Decitabine treatment was performed by adding 4 µM decitabine (A3656 Sigma-Aldrich) to the culture media for 24 h and 1 µM for the next 4 days. After 5 days of treatment, cells were harvested and lysed for RNA extraction.

### Statistical analyses

Data are presented as mean ± SDs. Pairwise comparisons were evaluated by the Mann-Whitney U test. Group comparisons were performed by using 2-way Anova, followed by multiple comparison tests.

All statistical analyses were performed using GraphPad Prism IX software. P-values lower than 0.05 were considered statistically significant.

## Results

### DNA methylation analysis in kidneys of cystinotic and wild-type (WT) mice

To investigate epigenetic changes in cystinosis, we conducted a genome-wide DNAm analysis on whole kidneys harvested from *Ctns* ^*−/−*^ mice at the age of 6 months, when kidney parenchyma lesions are still at an early stage (Nevo et al., 2010), and from age-matched WT mice. Analyses were performed using the Illumina Infinium Methylation Mouse BeadChip array, which covers 862,927 CpG sites. Following data preprocessing and quality controls, including bisulfite conversion efficiency, hybridization, extension, and staining, we obtained the distribution of DNAm levels in each sample, showing the expected bimodal pattern for both beta- and M-values (Supplementary Figure S1).

**FIGURE 1:**
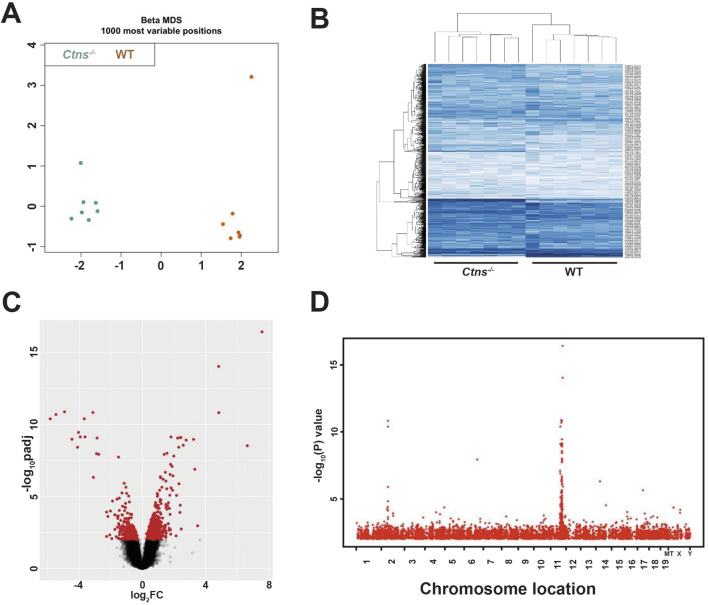
Analysis of DNA Methylome of 6-Month-Old *Ctns* ^*−/−*^ and WT mice Kidneys. **(A)** Multidimensional scaling (MDS) of the top 1000 most variable sites from 6-month-old WT and *Ctns* ^*−/−*^ kidneys. **(B)** Hierarchical clustering based on the top 1000 most variable sites, clearly separating *Ctns* ^*−/−*^ and WT kidneys in two distinct groups. **(C)** Volcano plot showing differentially methylated probes (DMPs), with the x- and y-axes showing the percent methylation difference and -log10 (p-value), respectively. Significant DMPs (methylation difference >15% and adjusted p-value <0.01) are shown in red, while non-significant DMPs are in black. **(D)** Manhattan plot of the statistically significant differentially methylated region. The x-axis represents the chromosomal location of the CpG position, and the y-axis shows the -log10 of p-value.

Normalized beta-values for each sample were then compared by means of multidimensional scaling (MDS) and Hierarchical clustering (HC) analyses to verify genome-wide DNAm levels among different sample groups. Results indicate that 6-month-old WT and cystinotic kidney samples clustered into two distinct groups (Figures 1A,B).

Linear modeling on M-values was used to identify differentially methylated positions (DMPs) (Figure 1C). DMPs were distributed across the entire genome, with occasional hot spots observed on chromosomes 2 and 11 (Figure 1D). Applying stringent criteria (|Δβ| ≥ 15% and adjusted p < 0.01), we identified in cystinotic kidneys 4,571 DMPs of which 673 (15%) were hypomethylated and 3,898 (85%) were hypermethylated, compared to WT kidneys.

**FIGURE 2:**
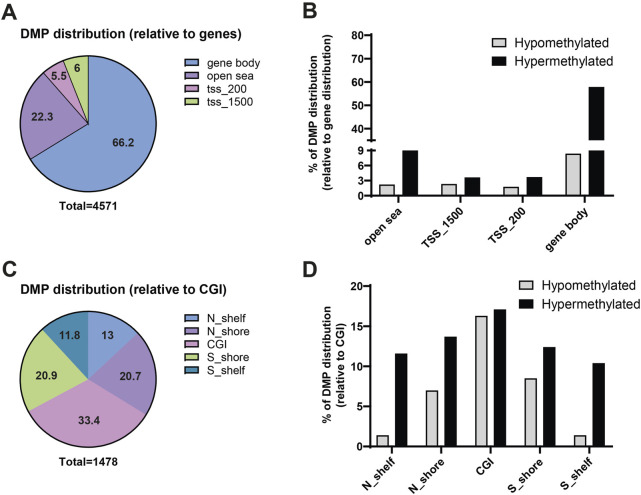
Genomic features of differential methylated positions (DMPs) in cystinotic mice kidneys. **(A,B)** The 4,571 DMPs were categorized based on their genomic location, including “open sea” regions, TSS_1500 (1,500 bp upstream of transcription start site), TSS_200 (200 bp upstream of transcription start site), and gene body. **(C,D)** Distribution of the 1,478 DMPs associated with CpG islands. N_Shelf: 4,000–2000 base pairs upstream of a CpG Island start site; N_Shore: 2000–0 base pairs upstream of a CpG Island start site. CpG_Island (CGI): CpG is within a CpG island; S_Shore: 0–2000 base pairs downstream of a CpG Island start site; S_Shelf: 2000–4,000 base pairs downstream of a CpG Island start site.

The DMPs were primarily located in the gene body (66.2%) and in non-coding (open sea) genomic regions (22.3%), with only 11.5% of DMPs located in promoter regions (Figure 2A). As reported in Figure 2B, hypermethylated DMP were localized mainly in the gene body (57.8%) and in the open sea (20%). Additionally, 1,478 DMPs were associated with CpG island (CGI) and were distributed as follows: 33.4% within a CGI, 20.9% in the S-shore, 11.8% in the S-shelf, 20.7% in the N-shore, and 13% in the N-shelf (See Materials and Methods for definition of N/S-shelf, N/S-shore) (Figure 2C). Hypermethylated DMP were enriched especially in N-shelf (11.6%), CGI (17.1%) and S-shelf (10.4%) (Figure 2D). Analysis of differentially methylated regions (DMRs) using the *ipDMR* algorithm identified 3,993 DMRs across 1,534 genes, with 84% of these regions resulting hypermethylated in cystinotic kidneys (Supplementary Table S1). Collectively, these results indicate a strong and extensive hypermethylation in the DNA of cystinotic kidneys.

**FIGURE 3:**
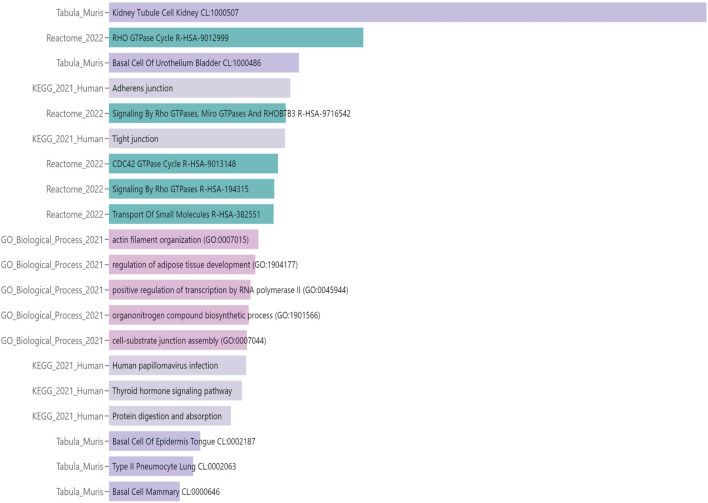
Functional annotation analysis of differential methylated positions (DMPs) in 6-month-old cystinotic mice kidneys. Gene-set enrichment analysis of pathways/ontologies associated with DMRs in 6-month-old *Ctns* ^*−/−*^ mice. The Enrichr-KG functional annotation tool was used to identify statistically significant functional associations, linking specific gene subsets to Biological Process categories [Gene Ontology (GO) or Signaling Pathways (KEGG pathway and Reactome databases) or Tabula Muris single-cell transcriptome data compendium].

Functional annotation of the DMPs was conducted through gene set enrichment analysis using Enrichr-KG (Evangelista et al., 2023). We included all the differentially methylated genes, regardless of CpG position. In addition, we analyzed both hyper- and hypomethylated genes, aiming to identify pathways affected by altered methylation, rather than focusing only on directionality or position of the alteration. The “Kidney Tubule Cell” (“*Tabula Muris*” annotation) was the most represented network, indicating that most of the epigenetic changes found in the cystinotic kidney occurred in tubular epithelial cells. Overall, the DNA methylation changes in cystinotic kidneys primarily affected genes encoding transporters and channels, cell signalling regulators, and proteins involved in cell-cell and transcriptional regulation (Figure 3; Supplementary Table S2). In line with functional enrichment analysis, which revealed that many differentially methylated genes were involved in the “Transport of small molecules”, we found that 74 out of 88 (84%) of differentially methylated genes in this pathway were hypermethylated (Supplementary Table S3).

### Methylation changes in *Ctns* ^*−/−*^ mouse kidneys reflect altered mRNA gene expression

To analyse the functional effect of the observed differences in DNAm levels, we performed qPCR analysis on kidneys homogenates obtained from 6-month-old WT and *Ctns* ^*−/−*^ mice, focusing on genes critical for kidney function.

**FIGURE 4:**
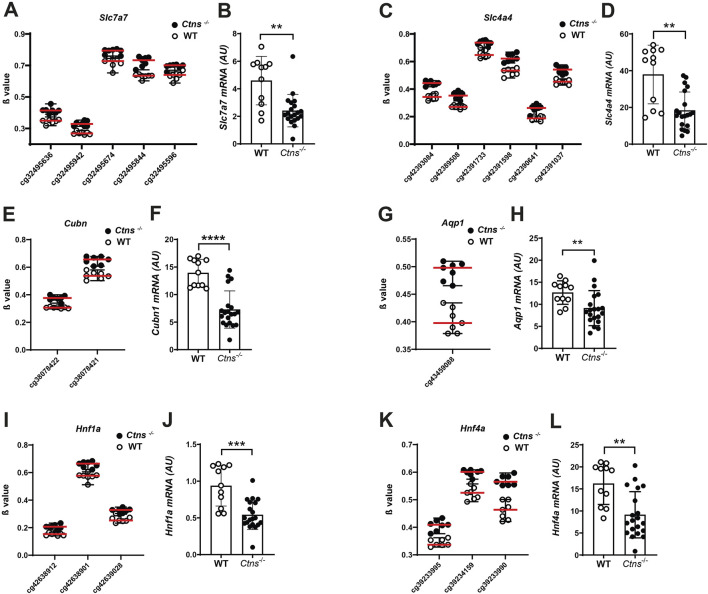
Gene expression analysis in 6-month-old mice kidneys. **(A,C,E,G,I,K)** β values of CpG associated to *Slc7a7*, *Slc4a4*, *Cubn*, *Aqp1*, *Hnf1a*, *Hnf4a* in 6-month-old *Ctns* ^*−/−*^ and WT mouse kidneys. **(B,D,F,H,J,L)**. *Slc7a7*, *Slc4a4*, *Cubn*, *Aqp1*, *Hnf1a*, *Hnf4a* mRNA levels were evaluated by qPCR analysis in whole kidneys from 6-month-old WT (n = 11) and *Ctns* ^*−/−*^ mice (n = 20). Results were obtained after normalization with the housekeeping genes *Hprt1* and are expressed as arbitrary units (AU). Differences between WT and *Ctns* ^*−/−*^ mice were analyzed using the Mann-Whitney U test. *p < 0.05; **p < 0.01; ***p < 0.001.

We analysed mRNA levels of *Slc7a7* and *Slc4a4,* two key members of the solute carrier (*Slc)* family. These genes encode the y + LAT1 transporter and the sodium bicarbonate cotransporter 1 (NBCe1), respectively. Consistent with the presence of five hypermethylated cytosines in *Slc7a7* and six in *Slc4a4*, both genes exhibited significant downregulation at the mRNA level in the kidneys of *Ctns* ^*−/−*^ mice, compared to WT (Figures 4A–D).

Additionally, we observed differentially methylated cytosines in *Cubilin (Cubn)* and *Aquaporin 1 (Aqp1)*, genes critical for kidney epithelial cell function, both of which are significantly downregulated in cystinosis (Raggi et al., 2014). We identified two hypermethylated cytosines in the *Cubn* gene that were associated by qPCR analysis with reduced *Cubn* mRNA expression in *Ctns* ^*−/−*^ kidneys, compared to WT controls (Figures 4E,F). Similar results were observed for the *Aqp1* gene (Figures 4G,H).

Additional analyses revealed hypermethylation of hepatocyte nuclear factor 1 (*Hnf1a)* and *Hnf4a* genes, which encode transcription factors crucial for proximal tubule function. We found three hypermethylated cytosines in each of these genes, with a corresponding strong downregulation of their expression in cystinotic kidneys (Figures 4I–L).

In summary, our findings demonstrate that differential methylation in cystinotic kidneys is associated with changes in the expression of numerous genes involved in kidney tubule function, including transporters and transcription factors.

### Demethylating treatment upregulates transporter expression in human conditionally immortalized PTEC

**FIGURE 5:**
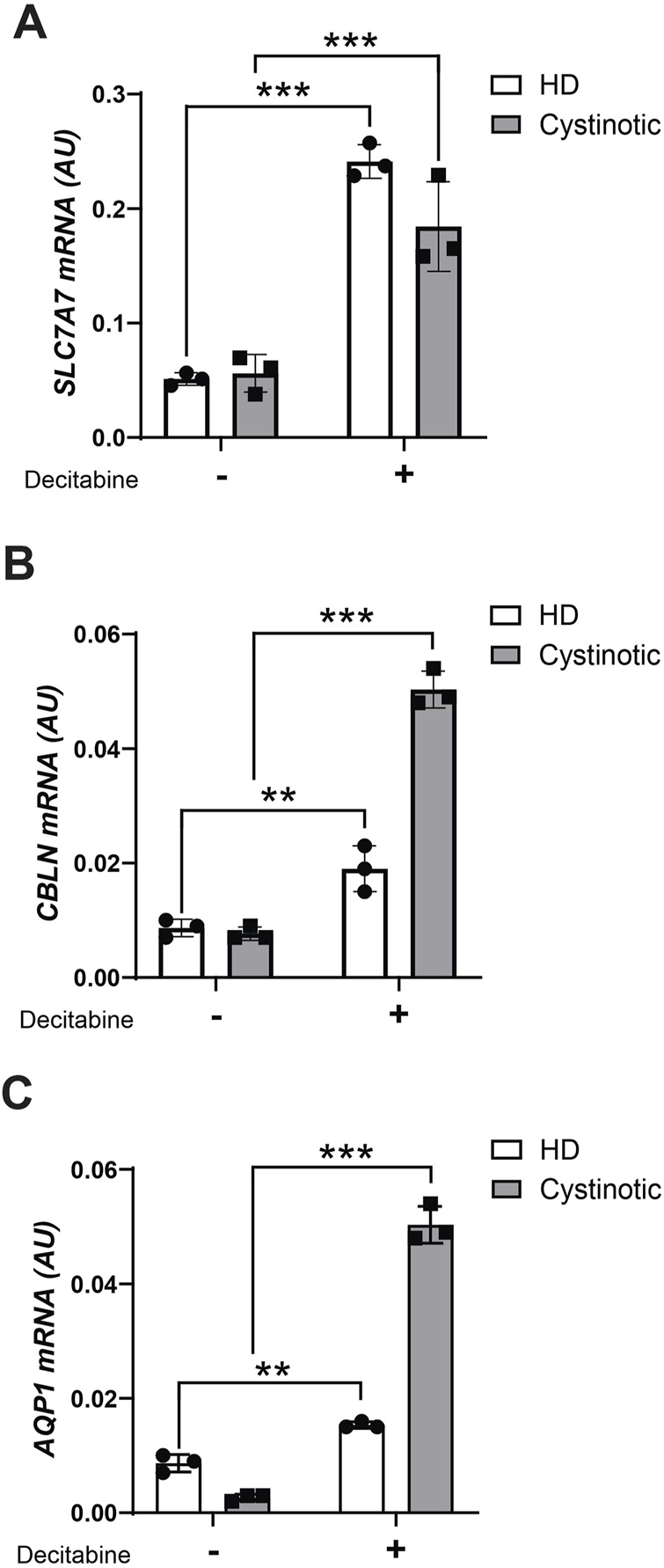
Decitabine treatment in ciPTEC. mRNA levels of *SLC7A7* **(A)**, *CBLN* **(B)** and *AQP1* **(C)** were evaluated by qPCR analysis in ciPTEC from a cystinotic patient (cystinotic) and a healthy donor (HD) treated (+) or not (−) with decitabine for 5 days. Results were obtained after normalization with the housekeeping gene *HPRT1* and are expressed as arbitrary units (AU). Differences between cystinotic and HD ciPTEC were analysed using 2-way Anova, followed by Sidak’s multiple comparison test, with a single pool variance. *p < 0.05; **p < 0.01; ***p < 0.001.

Based on the above results, we investigated whether this epigenetic signature could be reversed pharmacologically. To this end, we treated conditionally immortalized human proximal tubular epithelial cells (ciPTECs) from a healthy donor (HD) and a cystinotic patient (Wilmer et al., 2005) with the demethylating agent decitabine. At baseline, *AQP1* was the only transporter significantly downregulated in cystinotic ciPTECs compared to HD ciPTECs. However, decitabine treatment significantly upregulated *SLC7A7, CUBN*, and *AQP1* expression in both cell types (Figures 5A–C). Notably, the effect was more pronounced for *CUBN* and *AQP1* in cystinotic cells, suggesting that DNA methylation plays a key role in repressing these transporters in cystinosis and that demethylating agents may help restore their expression.

## Discussion

The *Ctns* ^*−/−*^ mouse is a well-established model for kidney disease in cystinosis (Nevo et al., 2010). Mice typically develop evidence of kidney damage, including tubular atrophy, inflammation, and interstitial fibrosis around 6 months of age (Nevo et al., 2010; Rossi et al., 2024). In this study, we have performed whole-genome DNAm profiling in 6-month-old kidneys to explore the potential role of epigenetic alterations in cystinosis-related kidney disease. Our results show significant epigenetic differences between *Ctns* ^*−/−*^ and WT mice, with a pronounced hypermethylation profile in cystinotic kidneys, even at this early stage of renal parenchymal damage. These methylation changes were primarily found in genes and pathways crucial for kidney function, particularly those related to the physiology of proximal tubules. Importantly, these methylation alterations correlated with changes in gene expression, as validated by qPCR analyses performed on key selected genes.

DNAm is a key epigenetic mechanism, and abnormal changes have been associated with a wide range of diseases, including cancer, and more recently, kidney diseases (Bechtel et al., 2010; Ko et al., 2013; Larkin et al., 2018; Marumo et al., 2015; Sagy et al., 2024; Smyth et al., 2014; Tampe et al., 2017; Tampe et al., 2015; Tampe et al., 2014; Yan et al., 2024). In our study, we found both hypermethylation (85% of DMP) and hypomethylation (15% of DMP) in cystinotic kidneys compared to WT kidneys. Both alterations, hypermethylation and hypomethylation, have potential effects in gene expression regulation and have been described in a wide range of pathologies spanning from cancer to neurodegenerative diseases (Ehrlich, 2009; Lu et al., 2013). Interestingly, our genome-wide methylation analysis showed that in *Ctns* ^*−/−*^ mice kidneys the majority of differentially methylated CpG sites were located within gene bodies (around 66%), while approximately 11% of DMP was found in promoter and enhancer regions. Previous studies have focused on CpG islands in promoter regions and their role in regulating gene expression (Fernandez et al., 2012; Nagae et al., 2011), but increasingly more studies are focusing on differentially methylated regions (DMRs) in gene bodies (Anastasiadi et al., 2018; Lokk et al., 2014), where fully methylated CpGs are more often reported (Bontha et al., 2017; Illingworth and Bird, 2009; Ko et al., 2013). Generally, promoter hypermethylation leads to heterochromatin formation, resulting in tightly packed DNA, reduced transcription factor accessibility, and gene silencing; DNA hypomethylation is often associated with gene activation or genomic instability (Illingworth and Bird, 2009). Conversely, gene-body methylation has been reported to positively correlate with gene expression, although it can also interfere with transcription elongation (Lokk et al., 2014). Increasingly, it is becoming clear that the relationship between gene-body methylation and mRNA expression levels is more nuanced and complex than initially thought. In our study, we cannot draw broad conclusions on the functional implications of gene-body hypermethylation in *Ctns* ^*−/−*^ kidneys based solely on location data. Nevertheless, when we sampled gene expression, we observed that hypermethylated regions, whether in gene bodies or promoter regions, were consistently associated with reduced mRNA levels. These findings underscore the importance of integrating DNA methylation and gene expression data and performing functional analyses to understand the precise molecular mechanisms underlying cystinosis-related kidney dysfunction.

Pathway enrichment analysis highlighted a significant enrichment of methylation changes in the “Kidney Tubule Cell” cluster, further emphasizing the critical role of tubular epithelial cells in the pathogenesis of kidney damage in cystinosis (Ivanova et al., 2023; Sur et al., 2024). Additionally, functional annotation revealed that many of the differentially methylated genes were predominantly involved in pathways related to “Transport of small molecules”, a process essential for maintaining renal function and electrolyte balance (Lewis et al., 2021). Strikingly, we found that 93% (55 out of 57) of differentially methylated *Slc* genes were hypermethylated. Accordingly, we found that key transporters such as *Slc7a7*, *Slc4a4*, as well as *Cubn* and *Aqp1* were not only hypermethylated but also significantly downregulated in cystinotic kidneys, suggesting that epigenetic silencing of transport-related genes could exacerbate tubular dysfunction. Similarly, in the kidneys of *Ctns* ^*−/−*^ mice, we found hypermethylation and reduced mRNA expression levels of transcription factors *Hnf1a* and *Hnf4a*, both critical for proximal tubule differentiation and function (Marable et al., 2018), further supporting the role of epigenetic modifications in disrupting tubular epithelial integrity. Indeed, loss of *Hnf1a* and *Hnf4a* expression has been implicated in the development of proximal tubular dysfunction (Marable et al., 2018), resulting in glycosuria and polyuria, which are hallmark features of cystinosis. Of note, Marumo et al. (2015) have observed that diabetes induces aberrant DNAm changes in proximal tubules, in particular in *Hnf4a* gene. Furthermore, recent findings emphasize the central role of DNAm in kidney-specific expression of amino acid transporters and of their master regulator, *Hnf1a* (Kikuchi et al., 2010). Consistently, our unpublished transcriptomic analyses of cortical kidney sections from 12-month-old mice revealed that 145 *Slc* genes were differentially expressed in cystinotic kidneys compared to WT mice, with 115 genes (79%) downregulated (data not shown). These findings strongly suggest that epigenetic dysregulation of transcription factors and transport-related genes plays a pivotal role in the pathogenesis and progression of cystinosis.

While the precise mechanisms underlying epigenetic dysregulation in kidney diseases remain unclear, emerging evidence points to oxidative stress and inflammation as key contributors (Elmonem et al., 2022; Ingrosso and Perna, 2020). Oxidative stress, a known driver of cystinosis-related tubular damage, has been shown to modulate DNAm patterns through alterations in methylation enzymes like DNMTs and TET proteins (Tampe et al., 2014). This interplay could amplify the hypermethylation observed in genes crucial for tubular cell function. Similarly, chronic inflammation, which characterizes cystinotic kidneys, can induce epigenetic changes that perpetuate inflammatory signaling and tubular injury. On these basis, it would be interesting to investigate whether interventions that have proven effective in preventing or delaying the progression of cystinosis, such as the ketogenic diet (Bellomo et al., 2024) or flavonoids (De Leo et al., 2024; De Leo et al., 2023), both known for their antioxidant and anti-inflammatory properties, exert at least in part, their effects by modulating the epigenetic landscape of cystinotic kidneys in mice.

The reversibility of DNA methylation represents an intriguing therapeutic opportunity for cystinosis. In our study, treatment of cystinotic ciPTECs with the demethylating agent decitabine successfully increased the expression of key transporters, including *SLC7A7*, *AQP1*, and *CUBN*. It is noteworthy that these genes were already transcriptionally active in HD cells, likely making them more responsive to upregulation upon treatment. Decitabine has been FDA-approved for the treatment of myelodysplastic syndrome and acute myeloid leukemia (Derissen et al., 2013) and, currently, several clinical trials on cancer are testing the efficacy of demethylating drugs (Wanner and Bechtel-Walz, 2017). Moreover, epidrugs are also effective to slow down the progression of renal fibrosis and inflammation in animals with progressive CKD (Tampe et al., 2017; Wanner and Bechtel-Walz, 2017) and decitabine treatment has been successfully used in mouse models of diabetic nephropathy (Larkin et al., 2018; Zhang et al., 2017). These findings highlight the therapeutic potential of epigenetic modulation to counteract gene silencing and improve tubular function. However, confirming the effect at the protein level and assessing downstream functional parameters would be essential to establish the real impact on cell physiology. Furthermore, an *in vivo* study using cystinotic mice treated with decitabine would be crucial to understand whether and how demethylation concretely affects disease pathogenesis.

In conclusion, this study provides compelling evidence that epigenetic alterations, particularly hypermethylation, contribute to cystinosis-related renal dysfunction. The identification of differentially methylated genes and pathways, especially those involved in tubular transport and transcriptional regulation, offers new insights into the molecular mechanisms driving disease progression. These findings pave the way for future research into the therapeutic potential of targeting DNAm to mitigate kidney damage in cystinosis. They also show that integrating epigenomic, transcriptomic, and proteomic data is essential to develop a comprehensive understanding of cystinosis-related kidney disease and to identify novel therapeutic targets for intervention.

## Data Availability

The datasets presented in this study can be found in online repositories. GEO accession number is GSE293586.
