# Supplementary material for: Genome-wide DNA methylation analysis identifies kidney epigenetic dysregulation in a cystinosis mouse model
Source: Front Cell Dev Biol. 2025 Aug 21;13:1638123. doi: 10.3389/fcell.2025.1638123 (PMC12408635; doi:10.3389/fcell.2025.1638123)
Supplement: Supplementary file 2 [file Table2.docx]

| Pathways | genes |
| --- | --- |
| Kidney Tubule Cell Kidney CL:1000507 | FOLR1, MEP1A, EPHX2, STARD10, SLC13A1, GPX3, CRYL1, CHPT1, SLC17A1, LDHB, DDAH1, IGFBP7, CISD1, SLC22A8, PAH, SLC4A4, ASS1, SLC22A18, GUCA2B, ME1, SLC27A2, SPP2, NDRG1, KCNJ15, DBI, TMEM174, GAS2, MIOX, PDZK1IP1, ALDOB, MSRA, SPP1, PRDX5, HGD, ATP1B1, CDA, and CELA1. |
| Reactome pathway RHO GTPase Cycle R-HSA-9012999. | ARHGEF28, TOR1AIP1, RHOBTB1, SH3RF1, DOCK9, ARFGAP3, ARHGAP11A, SLK, EMC3, WASL, RND3, RASAL2, LRRC1, MSI2, TRIP10, SPTBN1, CLTC, DOCK11, PLXNA1, TMPO, MCF2L, DIAPH2, ARHGAP10, EPHA2, DNMBP, WHAMM, ARHGEF39, DLC1, ABR, FNBP1, SRRM1, PARD6B, IQGAP2, PGRMC2, NCOA3, ACTN1, CAPZB, RHPN2, MYO6, TRIO, VAPB, CFTR, PTK2B, CDC42BPB, NISCH, STARD8, VAV2, ARHGAP24, REPS1, ARHGEF7, FAF2, ARHGEF25, FARP1, IQGAP1, PTPN13, VRK2, SYDE2, VAV3, ARHGAP26, STARD13, GRB2, STBD1, ARHGAP15, DOCK5, ARHGAP27, WDR91, UACA, DST, ARHGAP32, and DOCK6 |
| Basal Cell Of Urothelium Bladder CL:1000486 | PXN, JAG2, GMPR, TRPV4, GSR, KLF5, BCAR3, NET1, AGRN, LAMB3, TBC1D1, PHC2, RIPK4, PAPLN, RNF128, ITGB4, ITGA6, EZR, ATP1B1, ITGB6, SLC6A6, and EPHA2. |
| KEGG pathway Adherens junction. | INSR, PTPRF, RAC3, MET, PARD3, LMO7, ACTN1, ACP1, IQGAP1, ACTN4, EGFR, PTPRJ, CDH1, SORBS1, CTNNA1, IGF1R, FYN, WASL, and NECTIN1 |
| Reactome pathway Signaling By Rho GTPases, Miro GTPases And RHOBTB3 R-HSA-9716542. | TOR1AIP1, DNMBP, ABR, DOCK5, ARHGEF7, STBD1, PPP2R1B, CENPP, NCOA2, ARHGEF25, EMC3, PTPN13, ACTN1, PDPK1, FNBP1, CFTR, PAFAH1B1, RASAL2, PRKCZ, WASL, NISCH, SPTBN1, ABL1, PPP2R5E, TRIO, WHAMM, TMPO, SRRM1, ARHGAP10, LRRC1, ARHGEF28, RHPN2, FARP1, ARHGAP27, REPS1, RHOBTB1, VRK2, DYNC1LI1, STARD13, GRB2, ARFGAP3, EPHA2, MYH10, ARHGEF39, UACA, PTK2B, TRAK1, ARHGAP15, CDC42BPB, VHL, MSI2, STARD8, DIAPH2, VAV3, DOCK9, ARHGAP24, IQGAP2, ARHGAP26, ARHGAP11A, RND3, SRF, DLC1, SH3RF1, SYDE2, PGRMC2, ARHGAP32, IQGAP1, TRAK2, NCOA3, DOCK11, DOCK6, DST, VAPB, TRIP10, SLK, VAV2, PLXNA1, PARD6B, CAPZB, WDR91, CTNNA1, MCF2L, MYO6, FAF2, and CLTC |
| KEGG pathway Tight junction | EZR, MAP3K5, F11R, LLGL2, CFTR, ACTN4, ACTN1, MSN, EPB41L4B, PPP2R2A, SYNPO, CCND1, SLC9A3R1, TUBA1C, PPP2R1B, MYH10, JAM3, DLG1, MARVELD2, PRKCZ, PRKCE, PRKAA2, PARD3, PARD6B, PRKAG2, CGNL1, WHAMM, RUNX1, NEDD4L, MAGI1, MPDZ, and AMOT |
| Reactome pathway CDC42 GTPase Cycle R-HSA-9013148. | ARHGAP27, TMPO, TRIO, WASL, DLC1, VAV3, REPS1, ABR, FARP1, DOCK11, IQGAP1, ARHGAP24, DOCK9, CDC42BPB, ARHGEF7, STARD8, ARHGAP10, FNBP1, MCF2L, ARHGEF25, ARHGAP26, DOCK6, ARFGAP3, STARD13, VAV2, ARHGAP32, WDR91, DNMBP, and IQGAP2 |
| Reactome pathway Signaling By Rho GTPases R-HSA-194315 | ARHGAP10, SRF, MYH10, MCF2L, DLC1, DST, IQGAP1, STBD1, NISCH, ARHGEF7, SRRM1, DOCK6, IQGAP2, VRK2, DNMBP, ABR, RHOBTB1, PTPN13, DOCK11, WDR91, SYDE2, UACA, DOCK5, PTK2B, CDC42BPB, WHAMM, DIAPH2, CENPP, ABL1, VAV3, DYNC1LI1, RHPN2, VAPB, GRB2, PGRMC2, ACTN1, ARFGAP3, TRIP10, FAF2, PPP2R1B, CAPZB, EPHA2, VAV2, ARHGEF28, ARHGAP15, ARHGAP26, ARHGEF25, FARP1, SLK, TRIO, ARHGAP24, NCOA3, PDPK1, CFTR, PAFAH1B1, SPTBN1, CTNNA1, STARD13, ARHGAP32, SH3RF1, WASL, STARD8, MSI2, TOR1AIP1, NCOA2, DOCK9, PPP2R5E, FNBP1, PLXNA1, PRKCZ, PARD6B, RASAL2, MYO6, CLTC, ARHGAP11A, ARHGAP27, REPS1, ARHGEF39, EMC3, RND3, LRRC1, and TMPO |
| Reactome pathway Transport Of Small Molecules R-HSA-382551. | SLC47A2, SOAT1, MYO5B, AQP3, CAND1, ABCC6, SLC35A1, SGK1, SLC16A10, CLCN6, AP2A2, SLC35B3, SLC13A4, ATP11A, SLC9A2, CLTC, SLC43A2, ABCC3, SLC30A6, ATP2B1, SLC11A2, SLC30A1, ANO6, TRPV3, SLC22A4, APOC3, SLC5A10, SLC17A1, CLTA, TSC22D3, SLC24A4, ATP6V0A4, NEDD4L, ATP8A2, APOE, AQP7, SLC22A18, TRPM4, CFTR, TRPV1, ABCA9, SLC3A1, SLC15A2, SLC30A2, NIPA2, SLC9A8, SLC1A1, SLC20A1, SLC5A1, RUNX1, ATP1B1, SLC6A13, SLC2A12, SLC4A4, CUBN, SLC36A1, SLC24A1, NIPAL3, SLC2A9, DERL1, ABCC4, SLC22A8, SLC7A7, SLC7A8, TPCN1, SLC2A2, MAGT1, ATP2C2, TRPC3, ATP6V1B2, SLC6A6, ATP9A, SLC5A12, LIPC, SLC13A1, SLC16A1, TFR2, TRPV4, GNG5, MCU, ATP8A1, AHCYL2, PCSK5, PSMA6, PSMB7, NCEH1, SLC17A7, and SLC47A1 |
| Gene Ontology: biological process regulation of adipose tissue development (GO:1904177). | SORL1, SIRT1, KLF7, TRPM4, and SPI1 |
| biological process positive regulation of transcription by RNA polymerase II (GO:0045944). | GABPB2, NFE2L2, TFDP1, HNF1A, HOXD4, EPAS1, NR2F1, APP, RBPJ, MLLT10, E2F2, GATA6, ZFHX3, ABL1, SIRT1, NCOA3, CSRNP3, SKI, SKAP1, SSBP2, FOXO1, GATA3, GLIS3, MET, MAML3, FOXP3, RSF1, RORA, SMARCD1, TET3, SOX8, NOTCH4, KMT2A, KLF5, MTF1, IKBKG, SATB2, TFDP2, HIPK2, MED14, WWTR1, TNIP1, BCAS3, ETV5, PPP3CB, APLN, LMX1A, NR1H4, HDAC4, ACVR1, SPI1, SRF, MITF, NCOA2, EPC1, THRAP3, BMP6, CDK8, NR3C1, ZBTB38, AGRN, COPS5, ITGA6, E4F1, IKBKB, TCF12, KAT6B, SALL1, WASL, DAB2IP, GLIS1, MYO6, AUTS2, RXRA, OTX2, VEZF1, RARG, ZEB2, HNF4A, MED13, RREB1, LMO7, YBX1, LRP5, HLF, AKIRIN2, HOXB3, TCF4, GCM1, PRDM2, HNF1B, NFIA, KLF7, SMAD7, RXRG, VEGFA, RUNX1, CDK13, TFR2, EGFR, ESRRB, STAT1, SLC30A9, and LPIN2 |
| biological process cell-substrate junction assembly (GO:0007044). | ITGA6, ITGB4, DLC1, SORBS1, BCR, WHAMM, ARHGEF7, LAMB3, DST, TESK2, and ACTN1 |
| organonitrogen compound biosynthetic process (GO:1901566) | APOE, MOCS3, SGPP2, SLC25A15, STARD10, CERS6, HACD3, ELOVL6, LPIN2, ELOVL2, ASS1, DEGS2, MGST3, ARG2, VAPB, PAH, SPTLC1, B4GALT5, CHPT1, SAMD8, ESD, ALAS1, TNIP1, MOCS2, PCSK5, SPNS2, GSTT1, and PLPP3 |
| biological process actin filament organization (GO:0007015). | NRAP, MYO7B, DLG1, FAT1, RUFY3, NEDD9, RHOBTB1, RND3, TRPV4, SHROOM3, CGNL1, DMTN, MYO6, ENAH, WASL, FHOD3, WHAMM, VIL1, MCU, GAS2, SHROOM4, PLS1, RAC3, CORO7, MYO5B, BCAR1, MYO1C, and ACTN1 belong to the |
| KEGG pathway Thyroid hormone signaling pathway | PLCB1, FOXO1, NCOR1, DIO1, NCOA2, MED27, NCOA3, RCAN1, MED13L, ATP1B1, MTOR, RAF1, RXRA, MED14, CCND1, NOTCH4, NOTCH2, STAT1, RXRG, MED13, SLC16A10, PLCE1, and PDPK1 |
| KEGG pathway Tight junction | EZR, MAP3K5, F11R, LLGL2, CFTR, ACTN4, ACTN1, MSN, EPB41L4B, PPP2R2A, SYNPO, CCND1, SLC9A3R1, TUBA1C, PPP2R1B, MYH10, JAM3, DLG1, MARVELD2, PRKCZ, PRKCE, PRKAA2, PARD3, PARD6B, PRKAG2, CGNL1, WHAMM, RUNX1, NEDD4L, MAGI1, MPDZ, and AMOT |
| KEGG pathway Protein digestion and absorption | DPP4, MEP1A, COL18A1, ACE2, SLC7A8, SLC1A1, SLC16A10, COL12A1, COL4A3, SLC7A7, COL16A1, SLC36A1, ATP1B1, SLC3A1, COL5A3, COL27A1, COL6A2, COL4A1, COL6A3, and KCNK5 |
| From Tabula Muris: Basal Cell Of Epidermis Tongue CL:0002187 | WFDC2, APOE, LAMB3, TGIF1, SLC6A6, ANTXR1, ITGA6, IGFBP7, DST, GPC3, JAG2, AGRN, CSPG4, NEDD9, ITGB4, and SH3BGRL. |
| Basal Cell Mammary CL:0000646 | COTL1, PALLD, COL16A1, LGR4, ACTN1, RBPMS, TUBA1C, RUNX1, SORBS1, CDA, SYNPO, LAMB3, APOE, FERMT1, and EMID1. |
| Type II Pneumocyte Lung CL:0002063 | CFTR, LAMB3, ATP1B1, ARHGEF38, ANK3, EPN3, WFDC2, KCNJ15, PRSS8, MET, TINAG, MYO5B, CDH1, ETV5, and PTPRF. |
