# Supplementary material for: Genome-wide DNA methylation analysis identifies kidney epigenetic dysregulation in a cystinosis mouse model
Source: Front Cell Dev Biol. 2025 Aug 21;13:1638123. doi: 10.3389/fcell.2025.1638123 (PMC12408635; doi:10.3389/fcell.2025.1638123)
Supplement: Supplementary file 3 [file DataSheet1.pdf]

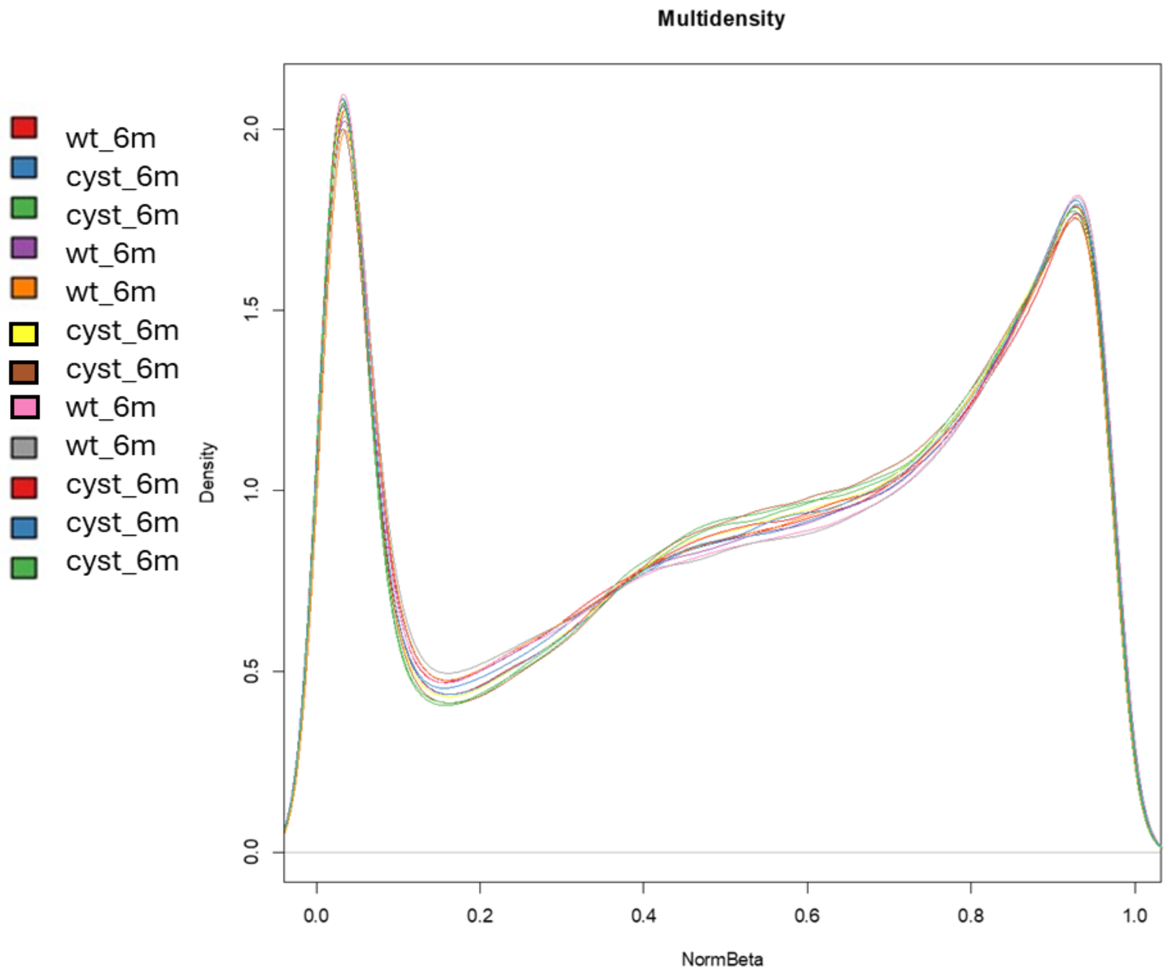

Supplementary Figure 1. Density distribution of β-values.

Density distribution of β-values of kidney from 6-months old cystinotic (cyst_6m) and WT (wt_6m) mice.
